# Safety and effectiveness of laparoscopic sacrocolpopexy as the treatment of choice for pelvic organ prolapse

**DOI:** 10.1080/2090598X.2019.1589781

**Published:** 2019-04-01

**Authors:** Sherif Mourad, Hisham El Shawaf, Ahmed Farouk, Hisham Abdel Maged, Amr Noweir, Bruno Deval

**Affiliations:** a Department of Urology, Ain Shams University Hospitals, Cairo, Egypt; b Geoffroy Saint Hilaire, Ramsay GDS, Paris V, France

**Keywords:** Female pelvic organ prolapse, laparoscopic sacrocolpopexy, laparoscopic sacrohysteropexy

## Abstract

**Objective**: To evaluate the safety and effectiveness of laparoscopic sacrocolpopexy (LSC)/laparoscopic sacrohysteropexy (LSH) at 1-year follow-up for female pelvic-organ prolapse (POP).

**Patients and methods**: In all, 52 patients were included and underwent LSC/LSH in the Eldemerdash Hospital, Ain Shams University, Cairo, Egypt. All patients with POP, with Grade ≥II of any anatomical site (anterior, posterior or combined) who were medically fit for general anaesthesia, were included in the study. Patients with previous major abdominal surgery, a body mass index (BMI) of >40 kg/m^2^ or un-correctable bleeding diatheses, were excluded. Preoperative data, peri- and postoperative functional and anatomical outcomes were assessed. The patients were followed-up at 3, 6 and 12 months postoperatively.

**Results**: Pre- and postoperative data were available for the 52 patients operated on for POP. The subjective cure rate was 92.3% and the objective cure rate was 98.1%. Failure was defined as recurrence of Grade ≥II POP.

**Conclusion**: LSC/LSH is a safe and effective procedure for the treatment of female POP due to its durable results and low rates of complications with high objective and subjective cure rates.

**Abbreviations**: BMI: body mass index; (RA)LSC: (robot-assisted) laparoscopic sacrocolpopexy; LSH: laparoscopic sacrohysteropexy; PFDI-20: Pelvic Floor Distress Inventory; PFIQ-7: Pelvic Floor Impact Questionnaire; POP: pelvic-organ prolapse; QoL: quality of life; SUI: stress urinary incontinence; TVM: total transvaginal mesh; VVP: vaginal vault prolapse

## Introduction

Since the introduction of the laparoscopic sacrocolpopexy (LSC) in 1994 [1], the advantages of this approach over both open and transvaginal techniques have been reported in many studies; with lower rates of recurrence, lesser incidence of dyspareunia, shorter postoperative hospital stay, and enhanced recovery time [2,3].

Providing minimally invasive access to the pelvis via a laparoscopic approach contributes to superior visualisation of the posterior part of the vaginal operative field and therefore improves the surgeon’s ability to avoid injury to nearby structures, such as the ureter and bladder, compared with the standard vaginal approach [4,5].

Our present study is a prospective clinical study to evaluate and assess the safety and effectiveness of LSC/laparoscopic sacrohysteropexy (LSH) as a treatment option for female pelvic-organ prolapse (POP). We report operative data, perioperative and early postoperative complications, and functional outcomes for this approach.

## Patients and methods

This is a prospective clinical study including 52 consecutive patients with POP using the same approach and technique. This study was conducted in the Department of Urology, Ain Shams University Hospital, Cairo, Egypt, from January 2015 to June 2017.

The surgical team had their original training on the laparoscopic technique in a tertiary referral centre (Geoffroy Saint Hilaire, Ramsay GDS, Paris V, France), where they were supervised by an experienced surgeon (B.D.; >650 cases) in the LSC technique. The operating team were trained on the setup for this technique in the same centre. The main study then started in Egypt using the same approach and technique. The surgical team included the first four surgeons who were supervised by the remaining two in the first 10 cases.

The mean (SD; range) age of patients was 56.44 (9.16; 37–76) years, 43 patients (82.7%) were married, two (3.8%) were divorced, and only seven were single. Five patients were menopausal. The mean (SD; range) body mass index (BMI) was 26.53 (5.38; 19.03–45.79) kg/m^2^.

### Inclusion criteria

Patients with POP Grade ≥II of any anatomical site (anterior, posterior or combined) who were medically fit for general anaesthesia and had the mental capacity to consent to the procedure and adhere to the follow-up protocol.

### Exclusion criteria

Previous major abdominal surgery, BMI of >40 kg/m^2^ and/or un-correctable bleeding diatheses.

### Ethical approvals

The Department of Urology, Faculty of Medicine Ethics Committee approval was granted before starting the study.

### Preoperative evaluation

Complete history and clinical examinations were performed to identify patient’s symptoms using both the Pelvic Floor Distress Inventory (PFDI-20), to evaluate frequency and severity of POP symptoms; and the Pelvic Floor Impact Questionnaire (PFIQ-7), to evaluate quality of life (QoL) secondary to POP, which gives a comprehensive assessment of the effect of pelvic floor disorders on the QoL of women [6,7] (Appendix). Clinical evaluation of the grade of the POP was performed using the Baden–Walker classification system [6,7] (Table 1). We agreed upon using this classification system instead of POP grading, which is recommended by the ICS, as we were more familiar with the former during our treatment of 18 patients (34.6%) with clinical stress urinary incontinence (SUI).

**Table 1.** Number of patients with POP in different compartments.

POP Grade	Cystocoele, *n* (%)	Uterine prolapse/VVP, *n* (%)	Rectocoele, *n* (%)
0	1 (1.9)	0 (0)	2 (3.8)
I	1 (1.9)	16 (30.8)	20 (38.5)
II	23 (44.2)	21 (40.4)	23 (44.2)
III	26 (50)	14 (26.9)	6 (11.5)
IV	1 (1.9)	1 (1.9)	1 (1.9)

All patients had routine preoperative blood and urine tests, and received an information sheet including the study details. The follow-up protocol was explained to every patient at least 1 week prior to consenting.

In all, 72 patients were diagnosed as having POP, either at the hospital clinic or through referral for further counselling on the feasibility of LSC. Five patients of the 72 declined surgery and were treated conservatively, either by pessaries or pelvic floor rehabilitation and exercises. Three patients were excluded due to being unfit for general anaesthesia. Four patients had multiple previous abdominal surgeries making the laparoscopic approach unsuitable for the procedure and another four were found to have a BMI >40 kg/m^2^. Of the remaining 56 patients who had the procedure, four were lost to follow-up, thus finally 52 patients were included in our present study for assessing the safety and effectiveness of the procedure.

### Operative technique

**Figure 1.**
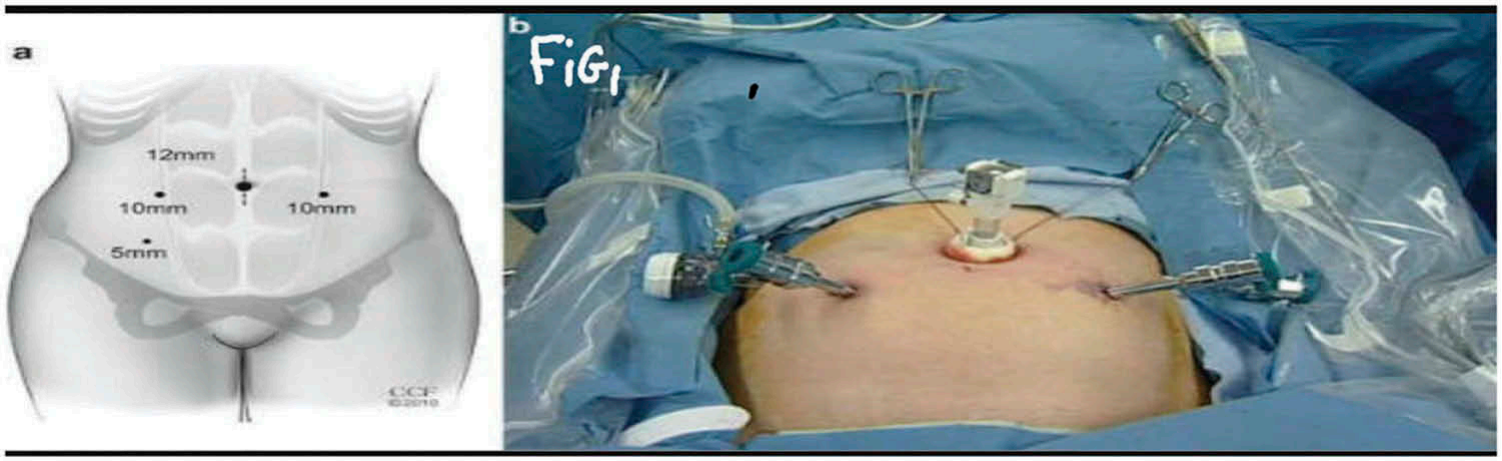
Port placement: (a) diagrammatic representation and (b) in a patient.

**Figure 2.**
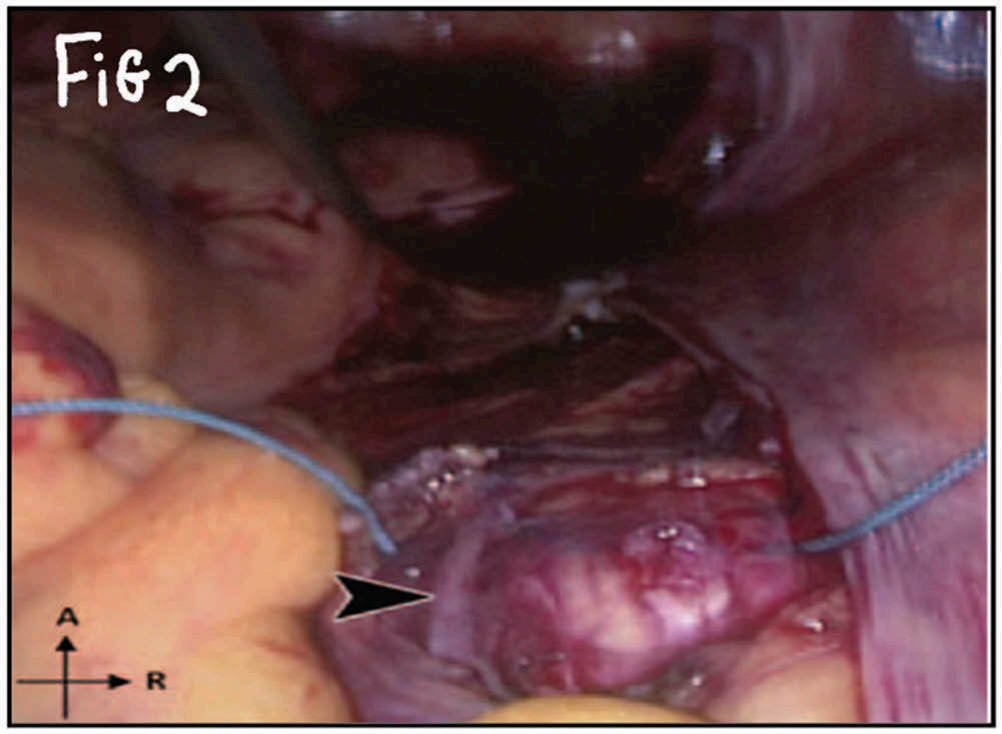
The first step is the dissection of sacral promontory.

**Figure 3.**
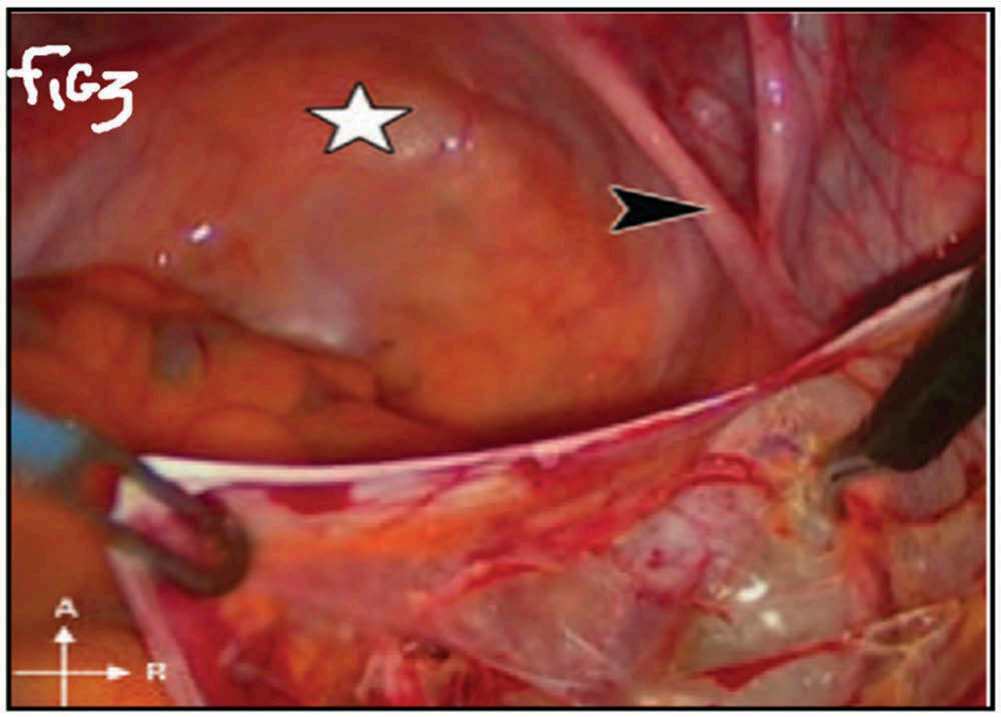
Dissection of posterior vaginal wall.

**Figure 4.**
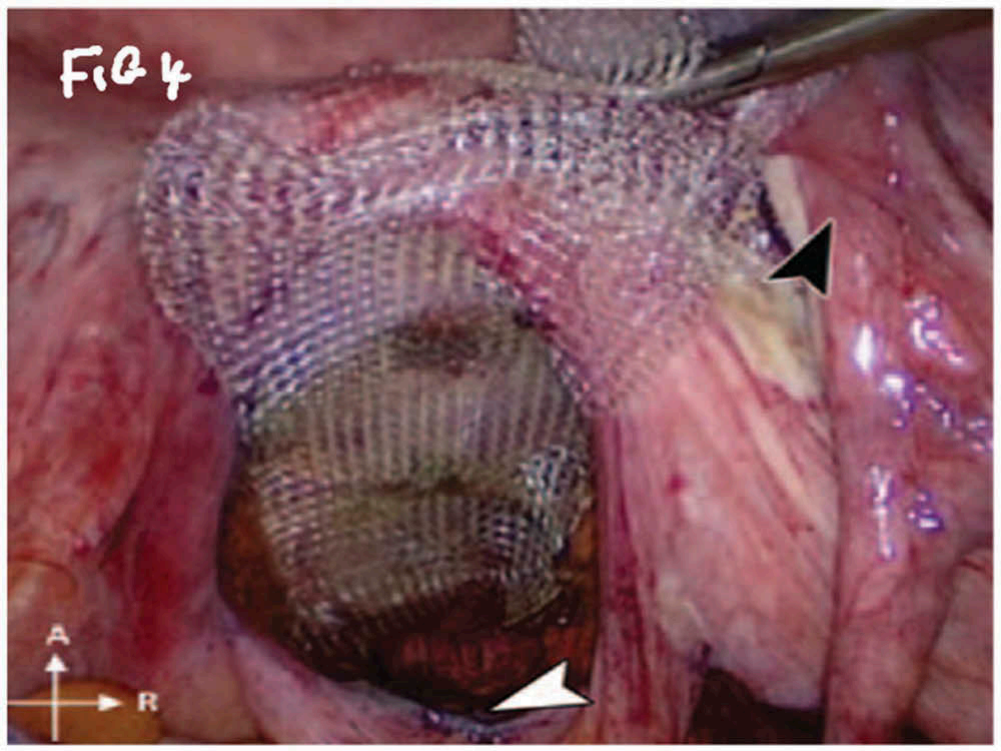
Fixation of posterior mesh.

**Figure 5.**
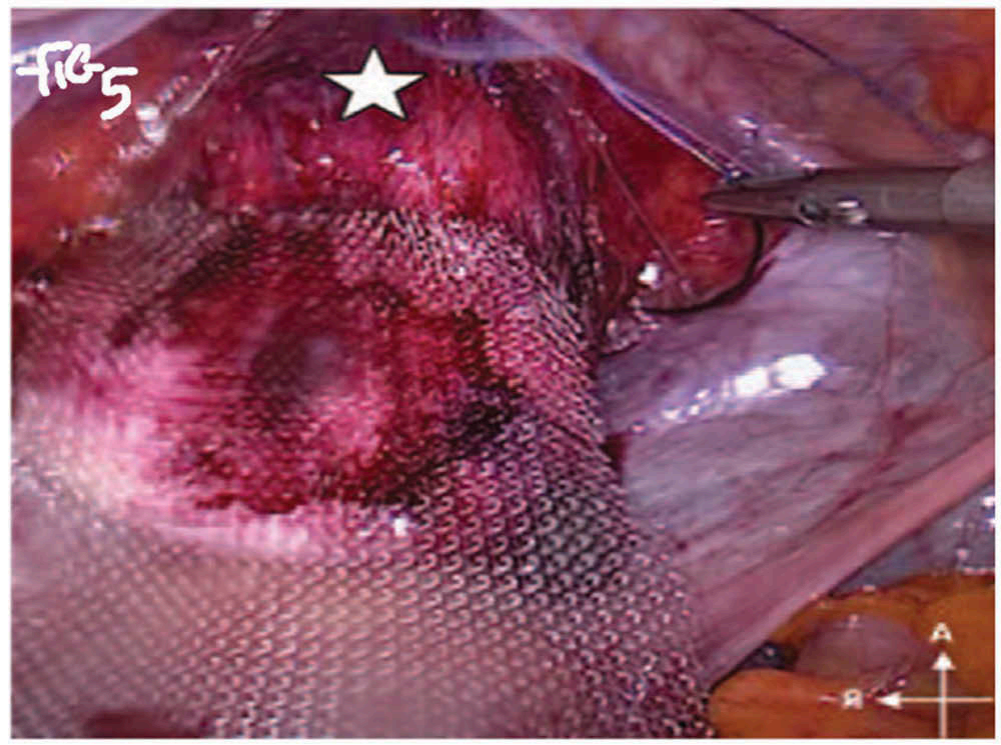
Fixation of anterior mesh.

**Figure 6.**
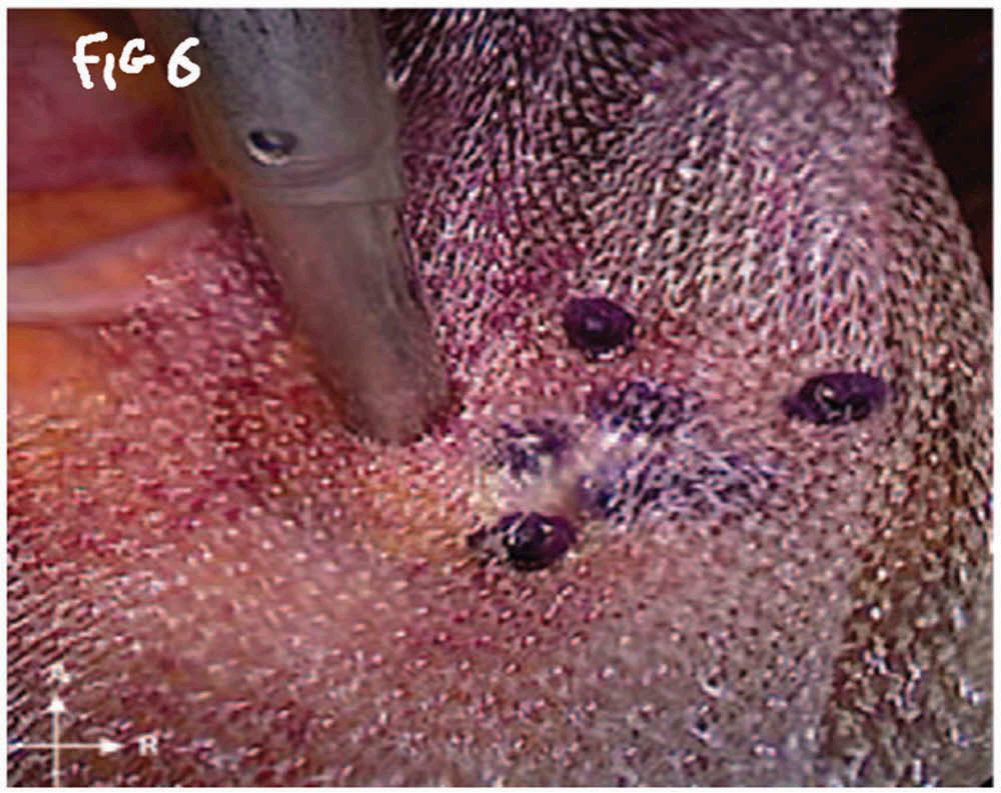
Anchoring mesh.

**Figure 7.**
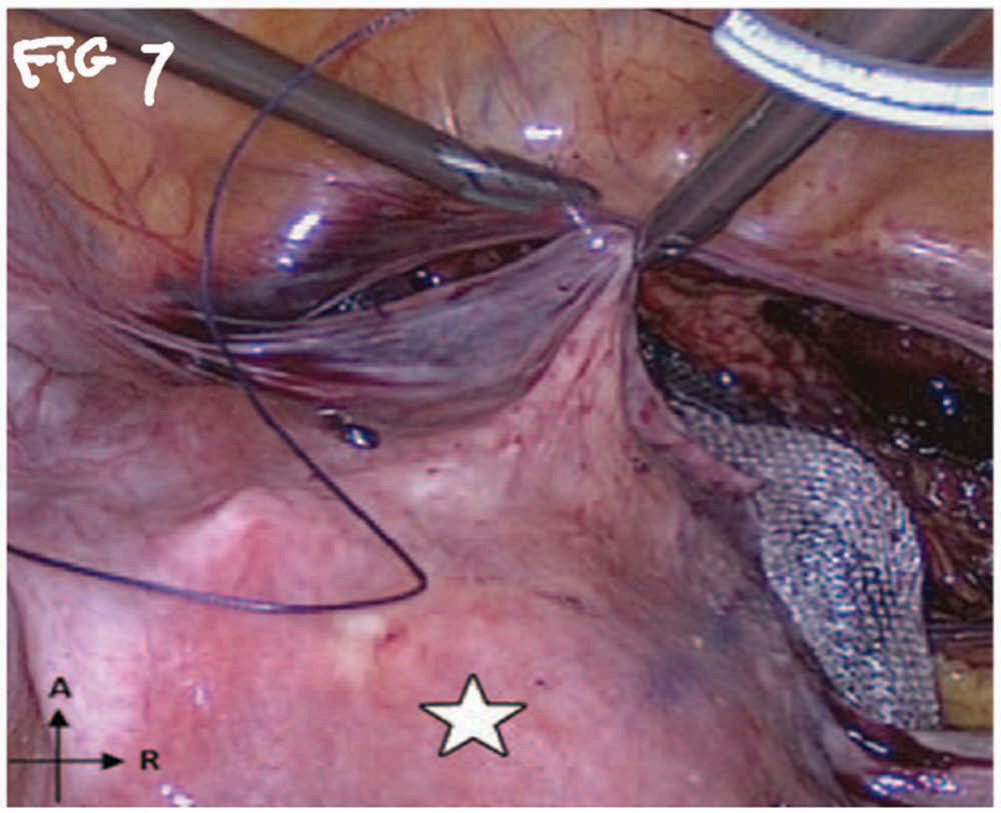
Closing of peritoneum.

All procedures were performed under general anaesthesia with the patient in supine Trendelenburg position. A uterine sound is placed in the uterus to manipulate the uterus and four transperitoneal ports (one 5-mm port, one 12-mm, and two 10-mm ports) are used (Figure 1). The first step is dissecting the sacral promontory and exposing the anterior longitudinal ligament (blue thread) and vein. A 2/0 polypropylene (Prolene®; Ethicon Inc., Somerville, NJ, USA) suture is taken transversely in the anterior longitudinal ligament (Figure 2). Then dissection of the right retroperitoneal tissue with incision of the peritoneum to enter the recto-uterine pouch and right uterosacral ligament. The posterior vaginal wall is stretched and pushed upwards by the malleable retractor and dissected carefully for ~6–8 cm downwards (Figure 3). Incision of the anterior peritoneum is done. Complete dissection of the anterior vaginal wall from the empty bladder is done with a malleable retractor stretching the anterior vaginal wall. Fixation of the posterior mesh (polypropylene mesh ~3 × 15 cm) is done using four to six 2/0 polyglactin 910 (Vicryl®; Ethicon Inc.) sutures to the posterior vaginal wall in front of the rectum (white arrow head) and right adnexa (Figure 4). The anterior mesh is divided incompletely into two limbs (right and left), leaving a common stem of ~5–6 cm. The anterior mesh common stem is fixed to the anterior vaginal wall with four to six sutures (Figure 5). The right limb of anterior mesh is passed through the right broad ligament and past the left limb of the anterior mesh through the left broad ligament. The two meshes are anchored on the sacral promontory using a tacking device (AbsorbaTack™; Medtronic, Minneapolis, MN, USA) (Figure 6). Reperitonalisation over the anterior mesh, posterior mesh and sacral promontory is done to avoid the risk of bowel adhesions and complications (Figure 7). We evaluated operative outcomes including: intraoperative complications, blood transfusion, associated surgical procedures, use of tacking device on the promontory, number of anterior and posterior meshes used, and operative time.

### Data collection and analysis

Operative data including: operative time, estimated blood loss, intra- and postoperative complications, and hospital stay were prospectively collected. Functional outcomes including *de novo* SUI, *de novo* dyspareunia were also recorded. For data analysis, we used the Statistical Package for the Social Sciences (SPSS®), version 16.0 (SPSS Inc., IBM Corp., Armonk, NY, USA).

In all the patients, follow-up data of ≥1 year were available. Outpatient visits were scheduled at 3, 6 and 12 months looking for evidence of recurrent POP, mesh erosion, SUI, and to complete the symptom questionnaires (PFDI-20 and PFIQ-7).

## Results

In all, 52 patients who underwent POP surgical repair by LSC/LSH had pre- and postoperative data available. The preoperative American Society of Anesthesiologists (ASA) grade of all patients ranged from I to III: 34 patients (65.4%) were ASA Grade I, 11 (21.2%) Grade II, and the remaining seven (13.4%) were Grade III.

In all, 16 patients (30.8%) had had previous abdominal surgeries (hernia repair, appendectomy, cholecystectomy, colectomy for colon cancer); five (9.6%) had had previous laparoscopic surgery. Seven of the 52 patients (13.5%) had undergone vaginal hysterectomy, and consequently were being treated for vaginal vault prolapse (VVP). The remaining 45 patients (86.5%) had uterine prolapse of different grades. Two patients (3.8%) had had caesarean sections and one patient (1.9%) had had tension-free vaginal tape inserted for SUI.

The mean (SD; range) operative time was 109.71 (75.85; 95–540) min. Anterior mesh was applied in all patients (100%) and posterior mesh in 50 (96.1%). Tacking on the promontory using the AbsorbaTack (5 mm) was used in 32 (61.5%) patients.

In all, 15 of the 18 patients (28.8%) with SUI preoperatively had symptom persistence, whilst the other three (5.8%) were cured by LSC. Three of these 15 patients (5.8%) underwent surgical re-intervention by transobturator tape (TOT). None of the patients developed *de novo* SUI or urge UI. One patient (1.9%) underwent TOT at the same time as LSC for treatment of SUI in response to the patient’s demands.

### Peri- and postoperative complications

Only two patients (3.8%) had intraoperative complications; one patient (1.9%) had a bowel injury and the second had a vascular injury. Both of them were managed intraoperatively with no long-term sequelae. The bowel injury patient had undergone previous colectomy and radiotherapy for colonic cancer. We found excessive intra-abdominal adhesions. Intraoperatively, primary laparoscopic repair was done, but on the second day postoperatively the patient developed acute abdomen, was explored, and intestinal resection anastomosis was done and the mesh was removed laparoscopically, and this case was considered a failure.

The vascular injury in one patient (1.9%), in the form of injury of the epigastric vessels, was ligated and controlled without further complications or need for blood transfusion.

There were no intraoperative ureteric or urinary bladder injuries. None of our patients required conversion to open surgery or required urgent blood transfusion during the procedure.

According to the Clavien–Dindo classification, the early postoperative period passed uneventfully in 51 patients (98.1%) with the need of only routine oral pain control (Grade I), except for the patient who had the bowel injury (Grade IIIb). He was returned to operating theatre on the second postoperative day for laparoscopic colectomy and re-anastomosis with removal of the meshes. Surgical re-intervention by TOT was performed in three patients (5.8%).

Mesh erosion was encountered in one patient (1.9%) and was managed conservatively with local oestrogen creams.

### Functional outcomes

The PFDI-20 and PFIQ-7 questionnaires were completed pre- and postoperatively, with both showing significant improvement in patient’s symptoms (Table 2).

**Table 2.** Mean scores for the PFDI-20 and PFIQ-7 questionnaires before and after LSC/LSH (Wilcoxon test).

	PFDI-20	PFIQ-7
Before surgery, mean (SD)	173.67 (58.41)	151.12 (48)
After surgery, mean (SD)	55.47 (37.49)	28.43 (32.57)
***Z***	6.266	6.275
***P***	<0.01	<0.01

In all, 15 of the 18 patients (28.8%) with SUI preoperatively had symptom persistence, whilst the other three patients (5.8%) were cured after LSC. Three of these 15 patients (5.8%) underwent surgical re-intervention by TOT. None of the patients developed *de novo* SUI or urge UI.

Two patients (3.8%) developed *de novo* dyspareunia, which was alleviated by the use of local lubricants and vaginal oestrogen cream, and three patients (5.8%) complained of *de novo* constipation.

Five patients (9.6%) complained of *de novo* backache, which was managed conservatively by analgesics and anti-inflammatory drugs. *De novo* backache was observed in obese patients, but in the data analysis there was no statistically significant relationship between BMI and developing backache.

The subjective cure rate was 92.3% in 48 patients and the objective cure rate was 98.1% in 51 patients. The success rate was consistent with the objective cure rate of 98.1%. Failure was defined by recurrence of Grade ≥II POP.

Failure of surgery was considered and defined by POP recurrence with Grade ≥II POP when found on clinical examination in any compartment, which was encountered in one patient. In the remaining 51 patients, POP was successfully treated and this persisted during the entire follow-up period, which was a minimum of 1 year for all patients, with a median follow-up of 1 year. Recurrence of POP was encountered in only one patient (1.9%) who had a Grade II cystocoele postoperatively.

There was a highly statistically significant difference between the postoperative median grade of POP following LSC when compared to median grade preoperatively (*P* < 0.01) (Table 3).

**Table 3.** Comparison between median grade of POP before and after LSC/LSH.

	Median (range) POP Grade			
	Before	After	*Z*	*P*
Cystocoele	III (0–IV)	0 (0–II)	6.32	<0.01
Uterine prolapse/VVP	II (I–IV)	0 (0–I)	6.36	<0.01
Rectocoele	II (0–IV)	0 (0–II)	6.16	<0.01

In all, 49 patients (94.2%), 35 (47.3%), and 29 (55.7%) had Grade II and III, cystocele, POP and rectocoele, respectively, at time of inclusion in our study. After LSC, in the 51 patients (98.1%) who had cystocoele and/or rectocoele, the postoperative Grade was 0/I, which persisted for the duration of the follow-up; consistent with the objective cure rate of 98.1%. None of our patients had uterine prolapse of Grade >I during the follow-up period. We found a highly statistically significant difference amongst all patients when compared to their preoperative status (*P* < 0.01; Table 4).

**Table 4.** Comparison between POP grades in different compartments before and after LSC/LSH (chi-squared test).

		Cystocoele, *n* (%)		Uterine prolapse/VVP, *n* (%)		Rectocoele, *n* (%)	
		Before	After	Before	After	Before	After
POP Grade	0	1 (1.9)	50 (96.2)	0	51 (98.1)	2 (3.8)	49 (94.2)
	I	1 (1.9)	1 (1.9)	16 (30.8)	1 (1.9)	20 (38.5)	2 (3.8)
	II	23 (44.2)	1 (1.9)	21 (40.4)	0	23 (44.2)	0
	III	26 (50)	0	14 (26.9)	0	6 (11.5)	0
	IV	1 (1.9)	0	1 (1.9)	0	1 (1.9)	0
Chi-squared		94.245		100.24		85.2	
*P*		<0.01		<0.01		<0.01	

This table shows that LSC definitely cured POP in all compartments.

## Discussion

Although open approaches to correct POP are more widely used in many developing countries, there are some surgeons using the laparoscopic technique to achieve similar outcomes. The steep learning curve and early discouraging outcomes can influence the adoption of the technique; therefore, we tried in our present study to identify prospectively the safety and effectiveness of this approach.

When comparing the different outcomes from our present study to different international studies using other techniques, we identified significant advantages of both a shorter hospital stay and less morbidity in our study group using a laparoscopic approach, which did not affect functional outcomes when looking at both objective and subjective parameters.

Our mean operative time (109.7 min) was comparable to the Bacle et al. [8] (97.4 min) series of 501 patients, whilst significantly better than that of Freeman et al. [9] (144 min) and Leruth et al. [10] (123 min).

Our intraoperative complications included a single case (1.9%) of bowel injury, which was treated by bowel resection and anastomosis on the second day postoperatively, and another case of vascular injury that was controlled intraoperatively by ligating the injured vessel. No bladder or ureteric injuries were encountered in our present study. None of the patients required blood transfusion or conversion to laparotomy during the primary procedure. Bacle et al. [8] reported five cases (1%) that needed conversion to open laparotomy due to difficult dissection, one (0.2%) case of bowel injury, and none of their patients required blood transfusion. They attributed their low rate of complications to the fixation of the mesh to the levator ani muscle and reperitonalisation.

Mustafa et al. [11] reported two patients (4%) with bladder injury, two (4%) needed conversion to laparotomy, and one (2%) required blood transfusion. They had no cases of bowel injury. Other studies have reported comparable complication rates to ours [12] and overall our present series points to a reasonably safe technique when compared to other studies.

In the Bacle et al. [8] series, the objective cure rate reached 88.5%, with 58 (11.5%) cases of recurrence (failure). The subjective cure rate (based on patient’s satisfaction and POP QoL questionnaires) was 86.4%. The average subjective cure rate (patient satisfaction) was 94.4%, which is higher than the objective cure rate found by clinical examination (92%). POP recurrence operations were required in 6.2% of the patients. In a series of 55 patients who underwent LSC, Leruth et al. [10] found that *de novo* SUI occurred in 13 (23.6%) patients with an objective cure rate of 100%. In the Thubert et al. study [13] the objective cure rate was 88.7% and the subjective cure rate was 69.3%.

Freeman et al. [9], in their series of 26 LSC patients, reported four (15.4%) cases of *de novo* constipation, three (11.5%) recurrences, two (7.7%) *de novo* dyspareunia, and an absence of mesh erosion. In the Maher et al. study [14], comparing LSC to total vaginal mesh (TVM) placement for VVP, the objective success rate was significantly higher for LSC when compared to TVM, at 77% and 43%, respectively (*P* < 0.001). Total vaginal length was unchanged in the laparoscopic arm and was significantly shorter in the TVM group (*P* < 0.001). The mean patient satisfaction, on a score range of zero to 100, was significantly higher in the LSC group, at a mean (SD) of 87 (21) as compared with 79 (20) in the TVM group (*P* = 0.002). Interestingly, no significant differences were detected between the groups in the QoL analysis. The most obvious difference between the groups that may account for the higher satisfaction in the LSC group were the re-operation rates, which were four-times higher for the TVM group compared to the LSC group (22% vs 5%), especially related to mesh erosion and contraction [14].

Freeman et al. [9] compared abdominal with LSC after 1-year follow-up of 26 patients who underwent LSC vs 27 patients who underwent open abdominal sacrocolpopexy. They concluded that the subjective outcome was 90% for the open group and 80% for the LSC group. There were improvements as regards blood loss, haemoglobin, and shorter hospital stay (*P* < 0.01) in the LSC group compared with the open abdominal sacrocolpopexy.

There are no data to support the use of robot assistance over conventional laparoscopy and there is no medical evidence that surgical outcomes differ for robot-assisted LSC (RALSC) compared to conventional LSC. The increased costs associated with robotic surgery compared to conventional laparoscopy place a significant burden on the healthcare delivery system [15,16]. In a study comparing the short-term functional outcomes obtained after LSC in 47 patients and RALSC in 20 patients, Seror et al. [17] concluded that whilst being equivalent to LSC in the short-term for functional outcome, RALSC was superior in terms of blood loss and strict operative time, but this time advantage was nullified when comparing overall operating room time.

One of the important aspects in patient selection for LSC is BMI. Thubert et al. [13], discussed this point in their study when they compared the feasibility and outcomes of LSC in obese vs non-obese women and this comparison revealed that anatomical results were comparable in the obese and non-obese groups: cure rate was 87.1% vs 91.6% (*P* = 0.60). In our study present, we found a statistically insignificant correlation between developing postoperative back pain and BMI.

Finally, LSC/LSH is a safe and effective procedure for the treatment of POP, due to its promising results and low rates of complications, with high objective and subjective cure rates. Whilst, long-term randomised multicentre studies are warranted to establish LSC as the first-line treatment option for POP.
